# Impact of traffic congestion on asthma-related hospital visits in major Texas cities

**DOI:** 10.1371/journal.pone.0311142

**Published:** 2024-09-26

**Authors:** Mei Yang, Tiankai Wang

**Affiliations:** Department of Health Informatics & Information Management, Texas State University, Round Rock, Texas, United States of America; AUM: American University of the Middle East, KUWAIT

## Abstract

Asthma is one of the most prevalent chronic conditions in the United States and is particularly sensitive to environmental changes in urban areas. While it is known that traffic congestion contributes to increased vehicle emissions and poorer air quality, its direct association with asthma incidence has not been thoroughly explored. This study aimed to address this void by analyzing 148 city-level observations from 2016 to 2020 in Texas, using data from the Texas A&M Transportation Institute and Definitive Healthcare. We investigated the association between traffic congestion, measured by the travel time index, and annual city-level asthma hospital discharges, while adjusting for refinery productivity, minority groups, and education levels through multivariate regression. Our findings revealed a significant positive correlation between the travel time index and asthma visits, indicating that higher traffic congestion is associated with increased hospital visits for asthma. This finding remains consistent across different models, regardless of whether control variables are included. For the control variables, we found that higher refinery productivity was linked to elevated risks of asthma-related hospitalizations, aligning with previous research findings. Although correlations with Black or African American and Hispanic or Latino populations, as well as those with less than a high school education, were not statistically significant, a positive trend was observed. These results emphasize the impact of traffic congestion on asthma prevalence and the necessity for targeted public health interventions and urban planning strategies.

## Introduction

Asthma is a major public health concern in the United States (U.S.) due to its high prevalence and substantial burden. In 2021, about 25 million people in the U.S. had asthma, which is 7.7% of the population [1]. This is an increase from 2001, when 7.4% of the population had asthma [2]. Asthma leads to missed school and work days, heavy healthcare costs, and even fatalities. In 2021, 3,517 people died from asthma [1]. Although asthma is incurable, avoiding known risk factors can effectively control and prevent asthma attacks.

Asthma is a chronic respiratory disease characterized by variable airflow obstruction, bronchial hyperresponsiveness, and airway inflammation [3]. Besides research on asthma’s biological mechanism, most prior asthma studies are under the theoretical framework of the environmental health theory, i.e., “the theory and practice of assessing and controlling factors in the environment that can potentially affect adversely the health of present and future generations” [4]. This theory emphasizes identifying environmental hazards, such as air pollutants, and assessing how people are exposed to these pollutants through inhalation, particularly asthmatics who may be more susceptible. The environmental health theory also recognizes that social factors like socioeconomic status can influence exposure and health outcomes. Risk factors associated with asthma include social determinants like place of residence, race/ethnicity groups, education, insurance, and income [5–11]. From an environmental perspective, studies have found that early life and short-term exposure to air pollution, notably from traffic, increased the risk of developing asthma and asthma-related emergency room visits and hospitalizations [12, 13]. Specific air pollutants can significantly impact respiratory health, exacerbating conditions like asthma in both children and adults [3, 14–16]. For example, exposure to particulate matter with a diameter of 10 micrometers or less (PM_10_) and ozone (O_3_) increased the risk of persistent asthma [14]. A systematic review showed a significant relationship between asthma and several traffic-related pollutants, including O_3_, nitrogen dioxide (NO_2_), carbon monoxide (CO), sulfur dioxide (SO_2_), PM_10_, and particulate matter with a diameter of 2.5 micrometers or less (PM_2.5_) [3]. Additionally, prenatal exposure to air pollutants, particularly to PM_2.5_, was associated with childhood asthma development [15, 16].

While there are various sources of air pollution, traffic remains a significant source of urban air pollution [17]. It is well known that traffic congestion increases vehicle emissions, including pollutants like NO₂, PM_10_ and PM_2.5_, CO, and volatile organic compounds (VOCs). In congested areas, these pollutants lead to ambient air pollution. People living near high-traffic areas are exposed to higher concentrations of these pollutants, which can exacerbate asthma and impair respiratory health [18–20].

Although previous studies have explored the impact of traffic-related air pollution on asthma prevalence, the direct link between traffic congestion and asthma is still underexplored. Additionally, it is noteworthy that only a limited number of these studies have undertaken a comprehensive analysis of both environmental and social factors in relation to asthma occurrence [11]. To address these research gaps, we investigate whether traffic congestion is associated with asthma-related hospital visits, adjusting for other environmental and social factors.

The objective of this study is to directly explore the association between traffic congestion and asthma-related hospital visits in major Texas cities. We selected Texas as the geographic setting for this study because of several reasons. First, Texan cities vary in environmental and demographic factors as the second largest and most-populous state in the U.S. For example, certain cities, such as Houston, experience more air quality issues [21]. Health disparities are more significant in some Texan cities, like El Paso [22]. These variations might interact with our research target, traffic congestion, and further influence asthma incident rates. Second, during the period of our study, from 2016 and 2020, Texas has experienced significant population growth [23], leading to increased urbanization. This population boom has considerably strained the existing infrastructure, resulting in exacerbating traffic congestion. Third, the occurrence of asthma can be influenced by pollutants emitted by refineries [24–26], making it an important variable to consider in our study. Texas, being the forefront of the petroleum industry in the U.S., houses thirty-two (32) operable refineries, and their productivity data are accessible for our research. In summary, Texas serves as a relevant and unique area of study for us due to its diverse cities, rapid urbanization, and data accessibility. We conducted a series of multivariate regression analyses to examine the relationship between traffic congestion and city-level asthma hospital discharges in Texas. Additionally, we adjusted for other environmental and social factors in the analysis, including refinery productivity, specific minority groups, and education levels.

## Materials and methods

### Data sources

To empirically conduct our research, we collected data from various sources to measure asthma-related hospital visits, traffic congestion, and control variables. First, we collected city-level data on asthma-related hospital visits in Texas from Definitive Healthcare, a subscribed healthcare data vendor. This database encompasses covers active U.S. hospitals, and includes comprehensive hospital information such as general, executives, clinical, financial detail. For this study, we only collected the asthma discharge number of each Texan hospital through its API (Application Programming Interface) by setting the downloading criteria for year, state, ICD-10, and patient type. Specifically, we downloaded the inpatient and outpatient discharge numbers for J45 (ICD-10 code of asthma) from Texas hospitals for the years 2016 to 2020. The raw data from the Definitive Healthcare was provided at the hospital level, with each observation representing the number of asthma discharges in Texas hospitals, including claims from emergency room visits in a given year. To align with the city-level traffic congestion data, we aggregated the raw hospital discharge data into city-level data based on cities. Following this grouping, each observation represented the asthma discharge numbers of hospitals in a city for a given year. Consequently, “*asthma visits*” was represented by the annual asthma discharges, measured in thousands, from cities in Texas.

We collected traffic congestion data from Texas A&M Transportation Institute. Desirable characteristics of traffic congestion measure include ease of communication, applicability across different geographical scales, comparability to a certain standard, the ability to be measured on a continuous scale, reliance on travel time data, and the capacity to describe highly congested conditions [27]. In this study, we adopted the travel time index, because it satisfies all these criteria and is widely employed in academic and other publications [28]. Vieira and Haddad noted that if the travel time index is calculated without considering changes in composition over time, the analysis results may be biased [28]. They suggested a weighted travel time index for congestion measurement. However, this approach has not been widely adopted by practitioners or academics and is not yet available for download. Currently, the travel time index remains the best and most accessible measure of congestion. The travel time index is the ratio of travel time in the peak period to the travel time under free-flow conditions. For example, a value of 1.3 indicates a 20-munite free-flow trip requires 26 minutes during the peak period. We combined various variables at the city level to match the raw congestion data, as Texas A&M Transportation Institute only provided data at the city level. Definitive Healthcare’s discharge data is available from 2016, while the traffic congestion data is available up to 2020. Therefore, our dataset covers 2016 to 2020, comprising 148 city-level observations.

Regarding the control variables, we gathered Texan refinery productivity data, measured in barrels per day, from the Arnold & Itkin website [29]. The demographic and educational variables are from the U.S. Census American Community Survey. These variables include the percentage of Black or African American, the percentage of Hispanic or Latino, and the percentage of the population with less than a high school education (Age ≥ 18). We collected U.S. Census annual data from 2016 to 2020 and merged them with asthma visits and traffic congestion data by city and year. Table 1 shows the descriptive statistics of the variables.

**Table 1:** Descriptive statistics of variables.

Variables	Minimum value	Maximum value	Average value	Medium value	Standard deviation
Asthma visits (in thousands)	0.302	51.194	8.733	3.887	11.179
Travel time index	1.040	1.350	1.131	1.100	0.075
Refinery productivity (barrel per day)	0	1199	97.010	0	264.854
Percentage of Black or African American	0.382	48.501	13.650	8.467	11.844
Percentage of Hispanic or Latino	14.387	95.549	43.942	35.051	23.204
Percentage of no high school diploma	7.010	33.539	16.957	15.771	5.783

### Regression model

We conducted a series of multivariate regression analyses in Stata 17.0 to examine the association between traffic congestion and asthma visits in Texas, adjusting for factors including refinery productivity, the percentage of Black or African American, the percentage of Hispanic or Latino, and the percentage of the population with less than a high school education. The equation of the regression model is represented as below:

$$Asthma_{visits}=\beta_{0}+\beta_{1}Traffic_{congestion}+\sum Controls+City\;FE+Year\;FE+\varepsilon$$ (1)

In this model, *Asthma*_*visits*_ represents the number of asthma-related hospital visits in Texas, while *Traffic*_*congestion*_ quantifies traffic congestion using the travel time index. The term *β*_0_ denotes the intercept, and *β*_1_ is the coefficient corresponding to traffic congestion. ∑*Controls* encompasses the sum of additional variables, such as refinery productivity, the percentage of Black or African American, the percentage of Hispanic or Latino, and the percentage of the population with less than a high school education. *City FE* and *Year FE* refer to city-specific and year-specific fixed effects, respectively, to control for temporal and city variations. *ε* represents the error term, accounting for any residual variation in asthma visits not explained by the model.

Fixed effects regression models are commonly used with longitudinal or panel data. They allow researchers to adjust for unobserved, unit-specific, and time-invariant confounders [30]. By assuming some characteristics remain constant over variables like time or location, these models help avoid omitted variable bias. In our regression model, fixed effects for city and year address unobserved time and location-invariant endogeneity by focusing solely on changes in variables within the longitudinal data.

### Visual analysis

In addition to the regression analysis, we also used thematic maps to examine the interaction between asthma visits and the travel time index. We first mapped the spatial distribution of both asthma visits and the travel time index to visually investigate the relationship between asthma occurrences and traffic congestion. We then selected the four most populous cities in Texas to better visualize the trends of asthma visits and the travel time index over time.

## Results

Table 2 shows the results of different models, controlling for environmental and sociodemographic variables. In the first model, which only included the travel time index, we observed a significant positive relationship between the travel time index and asthma-related hospital visits. This indicated that higher traffic congestion is associated with an increase in hospital visits for asthma. The visual analysis reveals that cities with higher travel time index values tended to have more asthma visits (S1 Fig). In 2020, the travel time index and asthma visits declined notably, particularly in the four most populous cities: Austin, Dallas, Houston, and San Antonio (S2 and S3 Figs). This supports our finding that traffic congestion significantly influences asthma-related hospital visits.

**Table 2:** Association between asthma visits, travel time index, and other factors.

Variables	Asthma visits Model 1	Asthma visits Model 2	Asthma visits Model 3	Asthma visits Model 4
**Travel time index**	**45.5049** ^***^	**45.5049** ^***^	**44.9717** ^***^	**44.8881** ^***^
(p-value)	(0.001)	(0.001)	(0.001)	(0.001)
**Refinery productivity**		**1.1640** ^***^	**1.0552** ^**^	**1.1202** ^**^
(p-value)		(0.000)	(0.024)	(0.013)
Percentage of Black or African American			0.1142	0.0984
(p-value)			(0.666)	(0.710)
Percentage of Hispanic or Latino			0.0705	0.0141
(p-value)			(0.783)	(0.955)
Percentage of no high school diploma				0.2050
(p-value)				(0.600)
Constant	-44.3392^***^	-44.3392^***^	-46.7887^***^	-48.0179^***^
(p-value)	(0.003)	(0.003)	(0.004)	(0.005)
Observations	148	148	148	148
Adjusted R-square	0.973	0.973	0.973	0.973
City FE	Yes	Yes	Yes	Yes
Year FE	Yes	Yes	Yes	Yes

City FE, city fixed effects; Year FE, year fixed effects.

* An asterisk next to a number indicates a statistically significant p-value: * p < 0.1

** p < 0.05

*** p < 0.01. The specific p-values of the regression coefficients are given in parentheses.

After adjusting for the refinery productivity factor in the second model, we found that the travel time index still maintained a significant positive association with asthma visits. Furthermore, refinery productivity was also significantly positively associated with asthma visits, suggesting that higher refinery productivity elevates the risk of asthma-related hospitalizations.

In the third model, we included the travel time index, refinery productivity, and demographic data on minority groups, including percentages of Black or African American and Hispanic or Latino populations. We found that the associations of the travel time index and refinery productivity with asthma visits remained significant and positive. Although we observed a positive association between asthma visits and the percentages of Black or African American and Hispanic or Latino populations, this association was not statistically significant.

Finally, when we included the percentage of the population with less than a high school education in the fourth model, the results remained consistent. The travel time index and refinery productivity continued to exhibit a significant positive association with asthma visits. On the other hand, we also found that the percentages of the Black or African American and Hispanic or Latino populations, as well as the percentage of the population with less than a high school education, exhibited a positive association with asthma visits, although these relationships were not statistically significant.

The economic magnitude of the result shows that when the travel time index in a Texas city increases (or decreases) by one unit, the average hospital asthma discharge will increase (or decrease) by 45 thousand. Because the range of the travel time index is only 0.3, it is unrealistic that the travel time index increases by one unit. In practical terms, a ten (10) percent increase (or decrease) in the travel time index will result in a 4.5 thousand increase (or decrease) in average hospital asthma discharge from a Texas city.

## Discussion

Urban planning and transportation studies reveal that the built environment, such as major roadways, significantly impacts public health [31–33]. Poor urban design and inadequate public transportation infrastructure often lead to greater reliance on personal vehicles [32], which can result in traffic congestion. Additionally, the design of urban environments often places residential areas near to major roadways, exposing residents to related pollutants [34, 35]. Research has shown that traffic-related air pollution is linked to an increased incidence of asthma [12, 14, 35–37]. Pollutants such as NO_2_ were positively associated with both asthma scores and incidence [35–37]. Other air pollutants including O_3_, CO, SO_2_, PM_10_, and PM_2.5_ have been linked to an increased risk of asthma-related emergency room visits and hospitalizations [12, 14]. As traffic congestion increases emissions, the related pollutants can trigger and worsen asthma symptoms [35, 36]. Consequently, people living near congested areas face a higher risk of asthma and more frequent hospital visits. Implementing urban design strategies, such as situating residential areas away from major roadways, can help reduce exposure to air pollution and lower asthma incidence. This is particularly crucial in cities like Austin, Dallas, Houston, and San Antonio, where traffic congestion is a major concern. Efforts to reduce traffic congestion have been found to decrease levels of air pollutants, leading to significantly lower rates of asthma events in both children and adults [38, 39]. However, these studies mainly focused on the impact of traffic-related pollutants on asthma and did not directly examine the impact of traffic congestion on asthma.

Compared to previous work, we found a direct association between traffic congestion and asthma, showing that traffic congestion is associated with an increased risk of asthma visits. Prior studies suggested that traffic-related pollutants affect asthma, indirectly supporting our finding. This is because traffic congestion increases emissions, contributing to air pollutants that trigger asthma incidence. However, it is important to note that there are multiple sources of air pollution, and traffic congestion is just one of them. Studying the direct association between traffic congestion and asthma allows us to avoid potential confounding variables related to air pollution in the analysis. Our finding not only confirms previous research but also provides an empirical example of how built environments, such as traffic congestion, influence asthma incidence. A core principle in the environmental health theory is preventing environmental hazards at the source. Based on the direct association between asthma and traffic congestion found in our study, the policy makers can adopt interventions aimed at controlling traffic load, such as the implementation of appropriate traffic-restricted zones [40], to mitigate both traffic congestion and asthma occurrences.

In the visual analysis, we observed a significant drop in traffic congestion in 2020 due to COVID-19. At the same time, asthma-related hospital visits also experienced a substantial decline in 2020 (S1–S3 Figs). This supports our argument that traffic congestion plays a key role in asthma-related hospital visits. The COVID-19 pandemic and associated restrictions, such as stay-at-home orders, work-from-home policies, and school closures, led to a significant decrease in vehicle trips and consequently reduced traffic volumes and congestion across various states in the U.S. [41–43]. In Texas, urban counties experienced a greater reduction in vehicle miles traveled compared to rural counties [43], contributing to the observed decrease in traffic congestion in 2020 compared to previous years in our study. Additionally, given the association between traffic congestion and asthma visits, the reduction in traffic congestion during the pandemic can also explain the observed decrease in asthma visits during the same period.

For the control variables analyzed in this study, we found that higher refinery productivity is associated with increased asthma visits. This finding is consistent with the findings in previous studies that living close to refinery areas and exposure to refinery emissions significantly impacts asthma prevalence and exacerbations [24–26, 44–46]. However, one limitation is that the refinery productivity data used in this study was collected in 2022. We assumed that refinery productivity remained consistent throughout our study period due to the lack of data from other years. Future investigation is needed to validate the finding once more refinery data becomes available. We also found a positive association between asthma visits and being Black or African American, Hispanic or Latino, or having less than a high school education. However, these findings are not statistically significant. The reason for the lack of statistical significance might be due to the different proxies in measuring observations’ ethnic and education characteristics. Prior studies used the individual patient’s demographics [5, 8–10]. However, in our study, Definitive Healthcare could not provide the individual demographic due to the confidentiality regulations. We had to use the city-level demographic percentages to proxy the demographic characteristics. Future studies with individual demographic information are needed to further confirm these findings.

A key contribution of our study is its direct examination of the association between traffic congestion and asthma-related hospital visits. It further examined previous findings linking traffic-related pollution to a higher risk of asthma incidence. Additionally, it provides empirical evidence that contributes significantly to the existing body of knowledge on how the built environment influences asthma incidence. Another contribution is the inclusion of other environmental and social factors in the analysis. It comprehensively analyzed how both environmental and social factors influence asthma occurrence. The findings of this study highlight the importance of addressing traffic congestion as a public health priority. They also inform future research to explore more deeply how transportation-related congestion and air pollution affect the health of vulnerable populations at different geographical levels.

This study has several limitations. One limitation is the absence of individual-level demographic data, such as age and sex information, for the asthma visits. The lack of this data restricts our ability to explore how these demographic factors might influence the observed associations. Consequently, the generalizability of our findings is limited, as we cannot fully account for the variability in risk across different demographic groups. Another limitation is the assumption of consistent refinery productivity throughout the study period due to limited available data. This assumption may introduce potential bias, as variations in refinery productivity over time could have affected air quality and, subsequently, the asthma outcomes we observed. The lack of detailed refinery data prevents us from accurately accounting for these potential fluctuations. Future investigations into different age and sex groups, such as separate child and adult asthma cohorts, may yield varying results regarding risk factors and could benefit from a more comprehensive set of control variables. This could provide more comprehensive insights into the impacts of traffic congestion on various demographic groups. Moreover, studies incorporating multiple years of refinery data and individual demographic information are needed to further validate and refine the conclusions drawn from this work. Additionally, the method of aggregating hospital-level asthma visit data to city-level data introduces a potential source of aggregation bias. This bias could affect the precision of our findings, as it may obscure variations within the city, leading to an overestimation or underestimation of true associations at more localized levels. To mitigate this issue in future research, it would be beneficial to conduct analyses at finer spatial resolutions, such as neighborhood or census tract levels, where data availability permits. Moreover, employing multilevel models could help account for variability at different geographic levels, thereby reducing the potential for bias introduced by aggregation. Finally, the findings in our study identified the association between traffic congestion and asthma visits rather than establishing causality. Future studies employing causality analysis to examine whether these factors cause asthma incidence or related hospital visits would be beneficial.

## Conclusion

This study investigated whether traffic congestion is associated with asthma-related hospital visits in major Texas cities, adjusting for other environmental and social factors. Findings from this study suggest that increased traffic congestion is a significant risk factor associated with an increased risk of asthma-related hospitalizations. The findings have implications for reducing asthma-related hospitalization and preventing asthma by addressing the risk at its source. We suggest that lower traffic congestion can decrease the corresponding emissions, which in turn can significantly reduce asthma-related hospitalizations. This emphasizes the need for strategic urban planning and public health interventions. Urban planners and policymakers should consider the factor of traffic congestion when designing city layouts and implementing environmental regulations. Additionally, the results can inform future research and raise community awareness about the health risks associated with traffic congestion.

## Supporting information

S1 Fig Asthma visits and travel time index in Texas from 2016 to 2020: (a) 2016; (b) 2017; (c) 2018; (d) 2019; (e) 2020. State and place boundary data are collected from US Census Bureau, Geography Division: https://www.census.gov/cgi-bin/geo/shapefiles/index.php.
(TIF)

S2 Fig Top four Texas cities: Travel time index from 2016 to 2020. (TIF)

S3 Fig Top four Texas cities: Asthma visits from 2016 to 2020. (TIF)
