# Supplementary figures and images for: Impact of traffic congestion on asthma-related hospital visits in major Texas cities
Source: PLoS One. 2024 Sep 26;19(9):e0311142. doi: 10.1371/journal.pone.0311142 (PMC11426448; doi:10.1371/journal.pone.0311142)

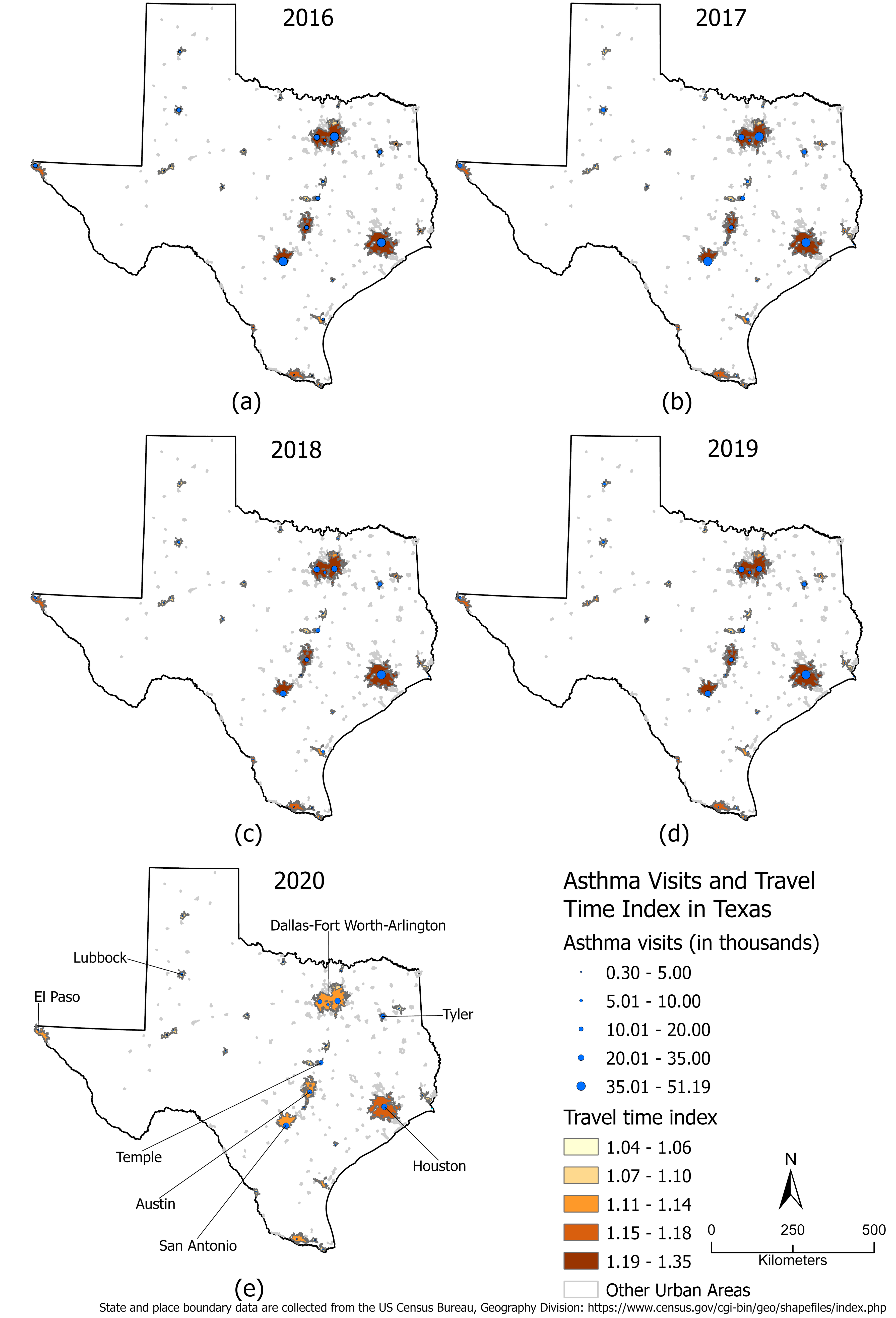

Supplement: S1 Fig — Asthma visits and travel time index in Texas from 2016 to 2020: (a) 2016; (b) 2017; (c) 2018; (d) 2019; (e) 2020. State and place boundary data are collected from US Census Bureau, Geography Division: https://www.census.gov/cgi-bin/geo/shapefiles/index.php. (TIF) [file pone.0311142.s001.tif]

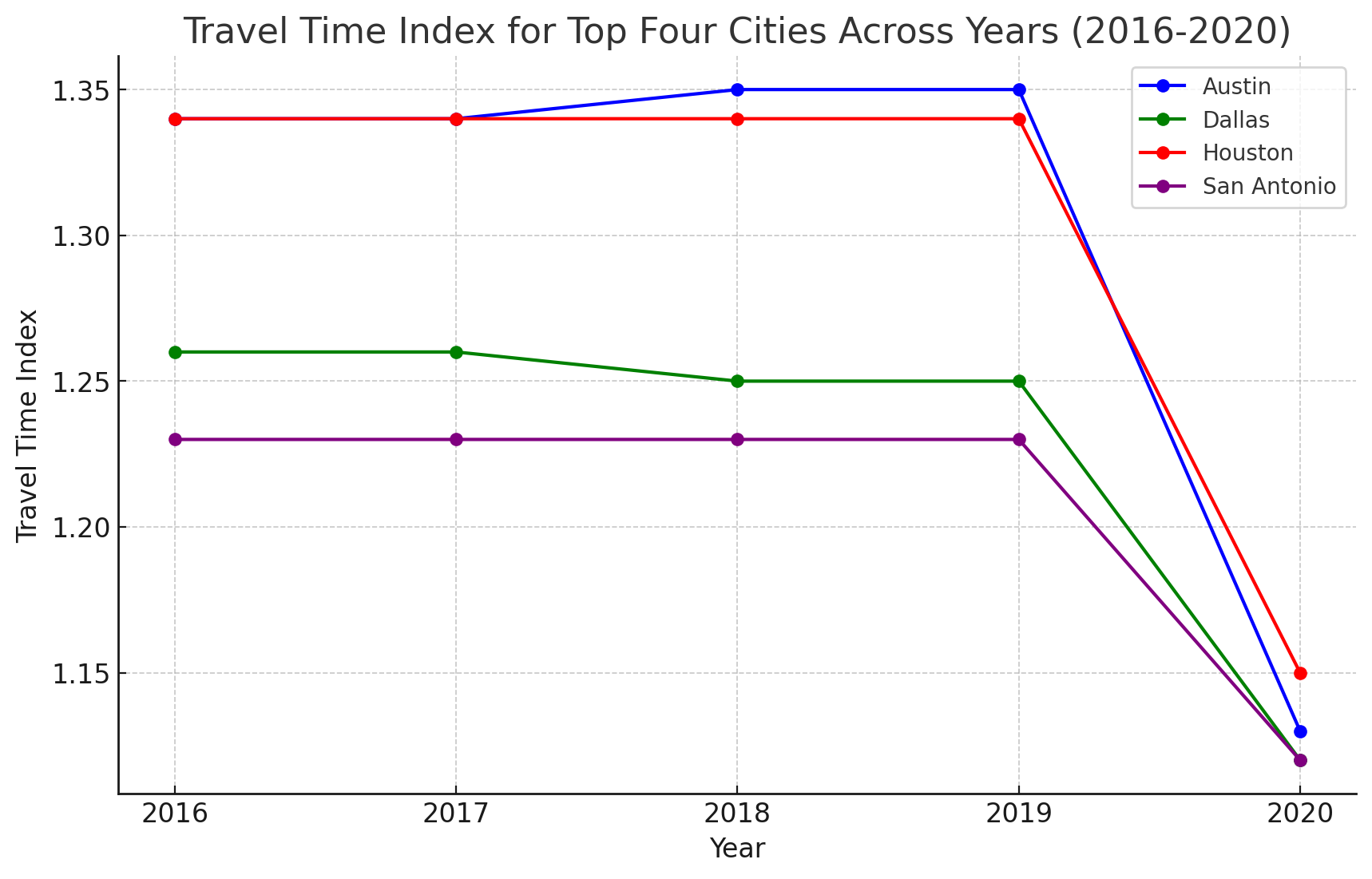

Supplement: S2 Fig — (TIF) [file pone.0311142.s002.tif]

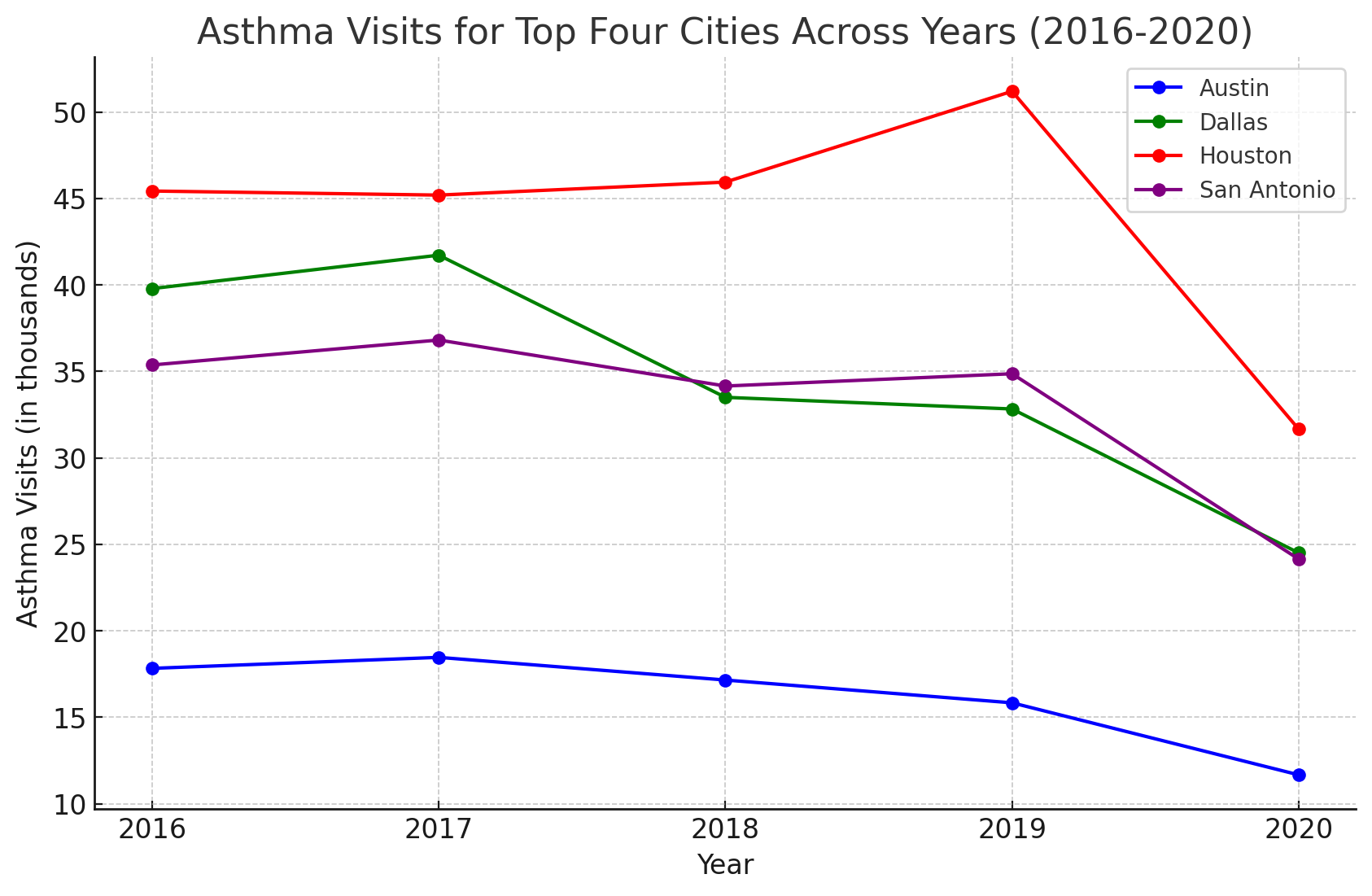

Supplement: S3 Fig — (TIF) [file pone.0311142.s003.tif]
